# Complement factors C4 and C3 are down regulated in response to short term overfeeding in healthy young men

**DOI:** 10.1038/s41598-017-01382-3

**Published:** 2017-04-27

**Authors:** Caroline Foghmar, Charlotte Brøns, Katrine Pilely, Allan Vaag, Peter Garred

**Affiliations:** 1 Rigshospitalet, Faculty of Health and Medical Sciences University of Copenhagen, Laboratory of Molecular Medicine, Department of Clinical Immunology, Section 7631, Copenhagen N, 2200 Denmark; 2 Rigshospitalet, Faculty of Health and Medical Sciences University of Copenhagen, Department of Endocrinology (Diabetes and metabolism), Copenhagen N, 2200 Denmark; 3 AstraZeneca Gothenburg, Mölndal, 43150 Sweden

## Abstract

Insulin resistance is associated with high circulating level of complement factor C3. Animal studies suggest that improper complement activation mediates high-fat-diet-induced insulin resistance. Individuals born with low birth weight (LBW) are at increased risk of developing insulin resistance. We hypothesized that high-fat overfeeding (HFO) increase circulating C3 and induce complement activation in a birth weight differential manner. Twenty LBW and 26 normal birth weight (NBW) young men were studied using a randomised crossover design. Insulin resistance was measured after a control-diet and after 5-days HFO by a hyperinsulinemic-euglycemic-clamp. Circulating C4, C3, ficolins, mannose-binding-lectin, complement activation products C3bc, terminal complement complex (TCC) and complement activation capacity were determined using turbidimetry and ELISA. HFO induced peripheral insulin resistance in LBW individuals only, while both groups had the same degree of hepatic insulin resistance after HFO. Viewing all individuals circulating levels of C4, C3, C3bc, TCC and complement activation capacity decreased paradoxically along the development of insulin resistance after HFO (P = 0.0015, P < 0.0001, P = 0.01, P < 0.0001, P = 0.0002, P < 0.0001, P = 0.0006). Birth weight did not influence these results. This might reflect a hitherto unrecognized down-regulatory mechanism of the complement system. More human studies are needed to understand the underlying physiology and the potential consequences of these findings.

## Introduction

Affecting more than 170 million people worldwide, diabetes constitutes a major threat to human health across the world^1^, with type 2 diabetes (T2D) accounting for more than 90% of all cases^2^. Individuals born with low birth weight (LBW) represent a distinct group of individuals with increased risk of developing insulin resistance and T2D due to different molecular and metabolic events during foetal life^3–5^. We have previously shown that adult LBW individuals have multiple metabolic defects and an unfavourable metabolic response to short term high-fat overfeeding (HFO) as compared to individuals with normal birth weight (NBW)^6, 7^.

Subclinical inflammation and improper immune activation may mediate or influence the impact of a high-fat diet on the development of insulin resistance^8^. Particularly the production of pro-inflammatory cytokines by dysfunctional adipocytes and infiltration of macrophages in adipose tissue is thought to induce insulin resistance, but also other immune cells may be involved^8–10^. Over the last decades, attention has been drawn to a role of the complement system as well. The complement system consists of tree distinct pathways of proteolytic cascades, namely the classical, alternative and lectin pathway. These pathways generate C3 convertases efficiently cleaving C3 into C3a and C3b^11^. This leads to the cleavage of C5 into C5a and C5b and the generation of the C5b-9 terminal complement complex (TCC). Both C3a and C5a are anaphylatoxins and mediate a variety of biological functions including chemotaxis and inflammatory responses via specific receptors^12^. Several of the complement proteins have been linked to obesity and the metabolic syndrome, and especially a high level of C3 has been shown to associate with insulin resistance, obesity and cardiovascular risk factors^13–17^. Several animal studies suggest that improper complement activation mediates high-fat-diet-induced insulin resistance through the action of the anaphylatoxins^18–20^, but still it remains unclear whether there is a causal link between complement activation and insulin resistance in humans.

In this study we hypothesized that high-fat overfeeding would increase C3 and induce complement activation in a potential different manner in people with and without LBW. Accordingly, we investigated circulating levels of C3 and the complement activation products C3bc and TCC after a control diet and after short term high-fat overfeeding in a group of healthy young men with either NBW or LBW. To further address the impact of HFO on the different complement pathways we measured serum levels of C4, lectin pathway initiators and the functional capacity of all three complement pathways separately after a control diet and after HFO. Finally we aimed to explore whether the association between C3 and insulin resistance may be due to an association to hepatic or peripheral insulin resistance.

## Results

### Baseline characteristics

When comparing the NBW and LBW subjects before the short term HFO the LBW subjects had significantly lower height (P = 0.003) and had increased levels of fasting blood glucose (P = 0.01), fasting serum insulin (P = 0.02), and fasting serum C-peptide (P = 0.04) as shown in Table 1 (previously published data)^7^. There was no difference in peripheral insulin sensitivity (M-value) between the two groups, but LBW subjects had increased hepatic IR index (P = 0.02). The two groups did not have differences in serum levels of C4, C3, MBL, Ficolin-1, Ficolin-2, Ficolin-3 or in plasma levels of C3bc and TCC prior to HFO. Also, there was no difference in the functional capacity of any of the complement pathways between LBW and NBW subjects (Table 1).

**Table 1:** Subject characteristics of NBW and LBW subjects (CON) and after high-fat overfeeding (HFO).

	NBW (n = 25)		LBW (n = 18)	
	CON	HFO	CON	HFO
Age (years)	24.6 ± 1.1	24.7 ± 1.1	24.2 ± 0.5	24.2 ± 0.5
Height (m)	1.83 ± 0.07	—	1.77 ± 0.05^##^	—
Weight (kg)	78.4 ± 9.3	78.6 ± 9.7	77.1 ± 11.3	77.1 ± 11.4
BMI (kg/m^2^)	23.3 ± 2.4	23.3 ± 2.5	24.6 ± 3.8	24.6 ± 3.8
Fasting blood glucose (mmol/l)	4.59 ± 0.47	5.05 ± 0.40***	4.97 ± 0.48^#^	5.18 ± 0.34*
Fasting serum insulin (pmol/l)	30.9 ± 14.7	43.4 ± 29.2*	41.7 ± 14.6^#^	44.7 ± 21.9
Fasting serum C-peptide (pmol/l)	408 ± 146	529 ± 260*	492 ± 116^#^	539 ± 172
M value (mg/kg FFM/min)	13.7 ± 2.3	13.3 ± 3.3	13.5 ± 3.2	11.9 ± 3.6*
Hepatic insulin-resistance index	68.7 ± 34.1	113.7 ± 61.5***	102.3 ± 50.8^#^	108.7 ± 55.5
Serum C4 (mg/ml)	0.22 ± 0.05	0.20 ± 0.06**	0.24 ± 0.05	0.22 ± 0.06*
Serum C3 (mg/ml)	1.12 ± 0.15	1.04 ± 0.13***	1.14 ± 0.12	1.05 ± 0.14**
Serum MBL (ng/ml)	941 ± 920	920 ± 898	1124 ± 1149	1083 ± 1286*
Serum Ficolin-1 (µg/ml)	0.32 ± 0.10	0.33 ± 0.14	0.29 ± 0.07	0.30 ± 0.08
Serum Ficolin-2 (µg/ml)	6.38 ± 2.15	6.21 ± 2.03	6.49 ± 1.85	6.24 ± 2.10
Serum Ficolin-3 (µg/ml)	27.1 ± 9.0	25.6 ± 7.6	26.4 ± 7.7	27.6 ± 6.6
Plasma Albumin (g/l)	44.9 ± 2.3	44.6 ± 2.6	43.8 ± 1.9	43.7 ± 2.2
Plasma C3bc (AU/ml)	8.56 ± 2.25	7.75 ± 2.66	8.32 ± 2.11	6.54 ± 1.45***
Plasma TCC (AU/ml)	0.52 ± 0.09	0.45 ± 0.20**	0.51 ± 0.13	0.35 ± 0.10***^#^
C3bc/C3-ratio	7.71 ± 1.92	7.46 ± 2.41	7.30 ± 1.70	6.24 ± 1.11*
Classical pathway TCC (%)	97.4 ± 7.7	92.4 ± 7.6**	100.7 ± 6.0	95.2 ± 7.3*
Alternative pathway TCC (%)	94.5 ± 11.0	87.5 ± 10.1**	98.5 ± 10.4	89.2 ± 9.6***
Lectin pathway TCC (%)	75.3 ± 47.8	67.8 ± 49.7*	74.4 ± 56.1	63.5 ± 56.2**

Data are means ± SD. AU, arbitrary units; CON, control diet; HFO, High-fat overfeeding; TCC, terminal complement complex. NBW vs. LBW: ^#^P ≤ 0.05, ^##^P ≤ 0.01. CON vs. HFO diet: *P ≤ 0.05, **P ≤ 0.01, ***P ≤ 0.001.

### Metabolic response to short term high-fat overfeeding

The metabolic response to five days of HFO is shown in Table 1 and has previously been described in details^7^. In short, the fasting blood glucose level increased significantly in the NBW (P = 0.0005) as well as the LBW individuals (P = 0.05) in response to HFO. In the NBW subjects there was an additional increase in fasting serum insulin (P = 0.02) and fasting serum C-peptide (P = 0.01) levels. The LBW individuals became significantly more insulin resistant in peripheral tissue in response to HFO (P = 0.03) whereas the NBW individuals selectively developed hepatic insulin resistance (P = 0.0002).

### Effects of short term high-fat overfeeding on serum levels of C4, C3 and lectin pathway initiators and plasma level of albumin

**Figure 1:**
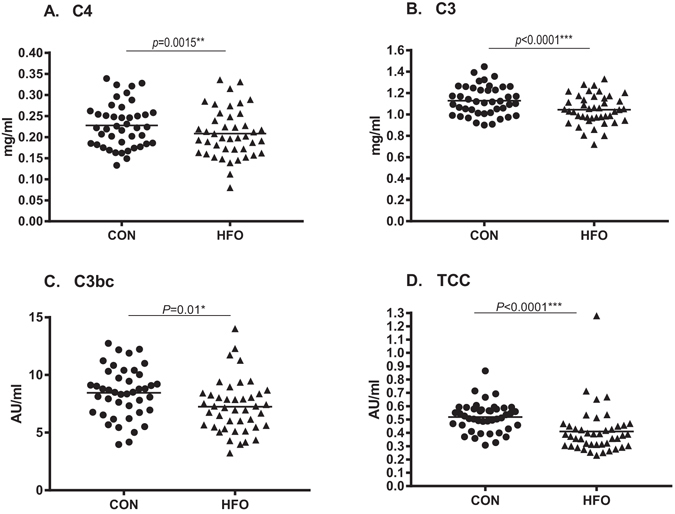
Circulating C4, C3, C3bc and TCC in response to overfeeding. Serum levels of C4 and C3 and plasma level of complement activation products C3bc and TCC after a control diet and after overfeeding in all subjects (n = 43). Data are reported as scatter-dot plots and medians. Paired two-tailed t-test p-values represent the difference in serum/plasma concentration before and after overfeeding. AU, arbitrary units; CON, control diet; HFO, high-fat overfeeding.

The serum levels of C4 and C3 decreased significantly (P = 0.0015 and P < 0.0001, respectively) in response to short term HFO when looking at the whole population (Table 2, Fig. 1), and also when looking at the NBW and LBW separately (Table 1). Birth weight category did not significantly influence the size of the decrease (data not shown). Serum concentrations of the lectin pathway initiators did not change in response to HFO in the whole population, but in LBW subjects MBL decreased significantly after HFO (P = 0.03). Plasma concentration of albumin did not change significantly after HFO in the whole population, or in NBW or LBW subjects.

**Table 2:** Impact of short term high-fat overfeeding on serum levels of C4, C3 and lectin pathway initiators, plasma levels of albumin and complement activation products C3bc and TCC and complement activation capacity in all individuals who completed the overfeeding challenge.

	n (43)		
	CON	HFO	Reference range
Serum C4 (mg/ml)	0.23 ± 0.05	0.21 ± 0.06**	(0.13–0.39)^#^
Serum C3 (mg/ml)	1.13 ± 0.14	1.04 ± 0.14***	(0.81–1.57)^#^
Serum MBL (ng/ml)	1018 ± 1013	988 ± 1066	—^##^
Serum Ficolin-1 (µg/ml)	0.31 ± 0.09	0.32 ± 0.12	(0.01–1.89)^36^
Serum Ficolin-2 (µg/ml)	6.42 ± 2.01	6.22 ± 2.04	(1.0–12.2)^37^
Serum Ficolin-3 (µg/ml)	27.0 ± 8.4	26.4 ± 7.2	(3–54)^38^
Plasma Albumin (g/l)	44.4 ± 2.2	44.2 ± 2.4	(40–53)
Plasma C3bc (AU/ml)	8.46 ± 2.17	7.25 ± 2.29*	(2.9–9)^###^
Plasma TCC (AU/ml)	0.52 ± 0.11	0.41 ± 0.18***	(0.3–0.7)^###^
C3bc/C3-ratio	7.54 ± 1.82	6.95 ± 2.05*	—
Classical pathway TCC (%)	98.8 ± 7.2	93.6 ± 7.5***	(69–129)^####^
Alternative pathway TCC (%)	96.2 ± 10.8	88.2 ± 9.8***	(30–113)^####^
Lectin pathway TCC (%)	74.9 ± 50.8	66.0 ± 51.9***	(0–125)^####^

Data are means ± SD. AU, arbitrary units; CON, control diet; HFO, High-fat overfeeding; TCC, terminal complement complex. CON vs. HFO diet: *P ≤ 0.05, **P ≤ 0.01, ***P ≤ 0.001. ^#^95 Percentile range from the manual of the SPAplus complement kit. ^##^Reference range left out due to the wide variations among healthy individuals. ^###^Unpublished internal laboratory reference ranges. ^####^± 2 SD reference range from the manual of WIESLAB Complement system screen.

### Effects of short term high-fat overfeeding on complement activation

Plasma level of C3bc, reflecting C3 activation, decreased significantly (P = 0.01) in response to HFO when looking at the whole population (n = 43) (Fig. 1) and LBW (P = 0.0008) but not in NBW (Table 1). Plasma TCC decreased significantly after HFO when analysing the whole population (n = 43) (Fig. 1) but also when analysing NBW and LBW subjects separately (p = 0.002 and p = 0.0004, respectively) (Table 1). The C3bc/C3-index decreased significantly after HFO when looking and the whole population (Table 2) as well as in LBW subjects (P = 0.03 and P = 0.02, respectively), but remained unchanged after HFO in the NBW subjects (Table 1).

### Effects of short term high-fat overfeeding on complement activation capacity

When looking at all individuals (Table 2), the capacity of the classical, alternative and lectin pathway decreased significantly in response to HFO (P = 0.0002, P < 0.0001 and P = 0.0006, respectively). This significant decrease persisted when viewing the two subgroups separately (Table 1). Birth weight was not related to the size of the complement activation decrease (data not shown).

### Reversibility of the complement dampening effect of short term high-fat overfeeding

**Figure 2:**
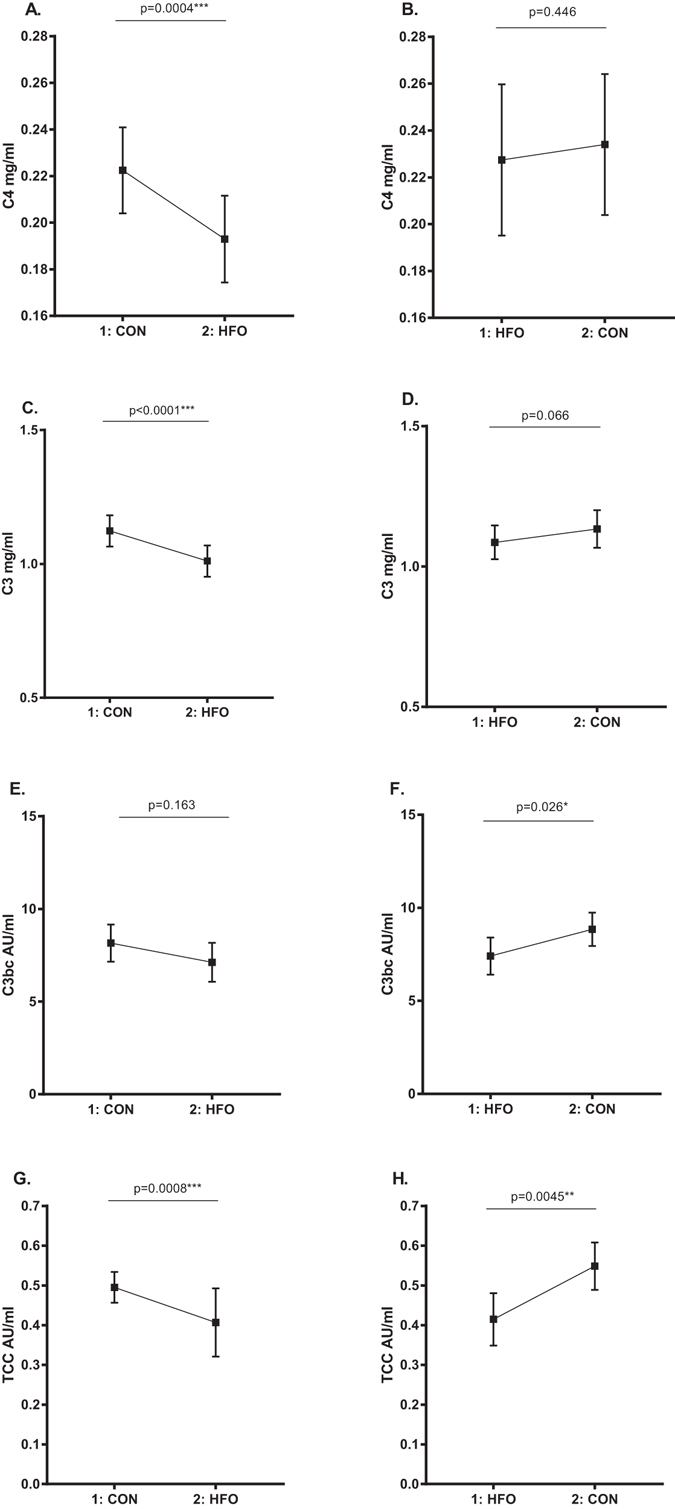
Reversibility of the impact of short term high-fat overfeeding on circulating C4, C3, C3bc and TCC. Comparison of the subjects that received the control diet first (n = 24) and the high-fat overfeeding diet 6–8 weeks after (diagram **A**,**C**,**E**,**G**), with the subjects that received the high-fat overfeeding first (n = 19) and the control diet 6–8 weeks after (diagram **B**,**D**,**F**,**H**). Data are reported as means with 95% CI. Paired two-tailed t-test p-values represent the difference in serum/plasma concentration of the different complement factors after control diet vs. after overfeeding. Please note the Y-axis benchmark in diagram (**A** and **B**) is 0.16, in diagram (**C** and **D**) 0.5, and in diagram (**E**,**F**,**G** and **H**) 0. AU, arbitrary units; CON, control diet; HFO, high-fat overfeeding; 1 = first study period; 2 = second study period.

**Figure 3:**
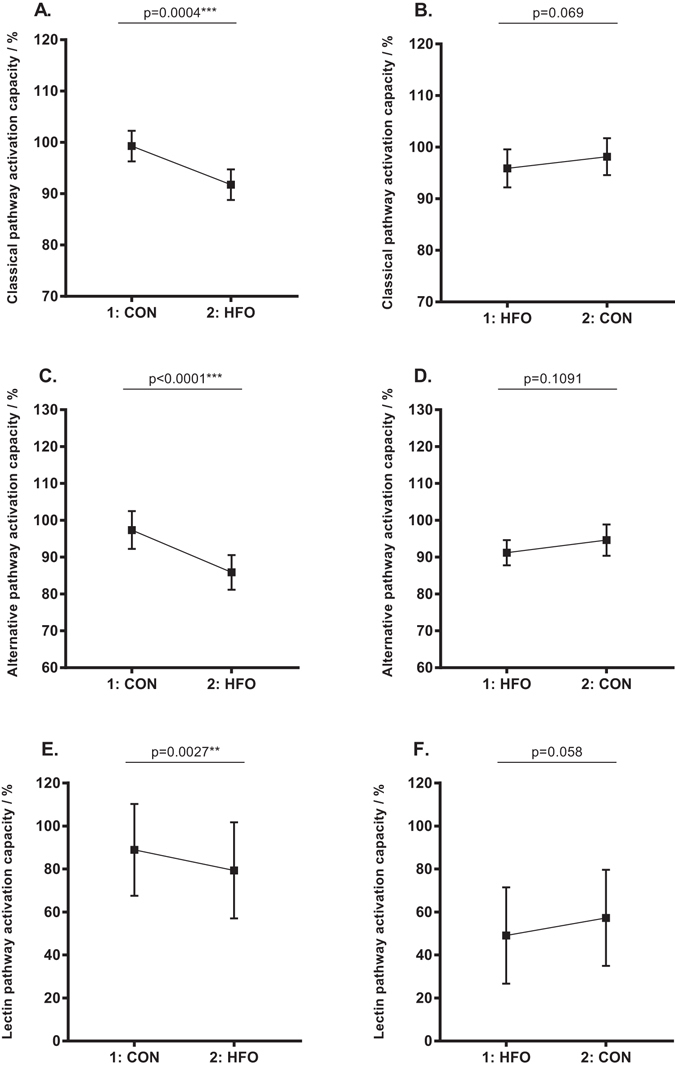
Reversibility of the impact of short term high-fat overfeeding on complement activation capacity. Comparison of the subjects that received the control diet first (n = 24) and the high-fat overfeeding diet 6–8 weeks after (diagram **A**,**C**,**E**), with the subjects that received the high-fat overfeeding first (n = 19) and the control diet 6–8 weeks after (diagram **B**,**D**,**F**). Data are reported as means with 95% CI. Paired two-tailed t-test p-values represent the difference in functional complement activation capacity of the different complement pathways after control diet vs. after overfeeding. Please note the Y-axis benchmark in diagram (**A** and **B**) is 70, in diagram (**C** and **D**) 60, and in diagram (**E** and **F**) 0. CON, control diet; HFO, high-fat overfeeding; 1 = first study period; 2 = second study period.

In order to examine if the complement dampening effect of HFO was transient or more profound we found, in the 24 individuals who received the control diet first followed by the HFO diet 6–8 weeks after, that C4, C3 and TCC decreased significantly after HFO (p = 0.0004, p < 0.0001 and p = 0.0008, respectively) (Fig. 2A,C and G), as did the functional capacity of the classical, alternative and lectin pathway (P = 0.0004, P < 0.0001 and P = 0.0027 respectively) (Fig. 3A,C,E). Viewing the 19 subjects whom received HFO first followed by the control diet 6–8 weeks after, C4 and C3 levels did not increase significantly after the control diet, although a trend was seen for C3 (P = 0.066) (Fig. 2D). C3bc and TCC increased significantly after the control diet (p = 0.026 and p = 0.0045, respectively) (Fig. 2F and H). The functional capacity of the classical, lectin and alternative pathway did not increase significantly, but a trend towards increase was seen (P = 0.069, P = 0.1091 and P = 0.058, respectively) (Fig. 3B,D and F).

### Association of hepatic insulin resistance with serum levels of C4, C3 and lectin pathway initiators

Taking all subjects into account (Table 3) we found a positive correlation between serum level of C3 and hepatic insulin resistance index (hepatic IR-index) on the control diet (CON) and after HFO (CON: r_s_ = 0.53 P = 0.0002, HFO: r_s_ = 0.43 P = 0.004). C4 was positively correlated with hepatic insulin resistance, but only on the control diet (r_s_ = 0.36 P = 0.02). When looking at the two subgroups (Table 3), serum level of C3 was still significantly correlated with hepatic insulin resistance in the NBW subjects regardless of the diet (CON: r_s_ = 0.51 P = 0.01, HFO: r_s_ = 0.41 P = 0.04). In LBW subjects C3 was correlated with hepatic insulin resistance on the control diet (r_s_ = 0.58 P = 0.01), and the same trend was seen after HFO but was not significant (r_s_ = 0.41 P = 0.09).

**Table 3:** Associations between hepatic IR-index and serum levels of C4, C3 and lectin pathway initiators.

Parameter	ALL (n = 43)				NBW (n = 25)				LBW (n = 18)			
	CON		HFO		CON		HFO		CON		HFO	
	r_s_	p value	r_s_	p value	r_s_	p value	r_s_	p value	r_s_	p value	r_s_	p value
C4 (mg/ml)	0.36	0.02^*^	0.08	0.59	0.34	0.09	0.03	0.89	0.17	0.49	0.18	0.46
C3 (mg/ml)	0.53	0.0002^***^	0.43	0.004^**^	0.51	0.01^**^	0.41	0.04^*^	0.58	0.01^*^	0.41	0.09
MBL (ng/ml)	−0.10	0.54	−0.11	0.46	−0.42	0.04^*^	−0.06	0.002^**^	0.30	0.22	0.42	0.08
Ficolin1 (µg/ml)	0.24	0.13	0.20	0.19	0.37	0.07	0.12	0.55	0.18	0.47	0.23	0.37
Ficolin2 (µg/ml)	0.14	0.38	0.21	0.18	0.06	0.78	0.23	0.27	0.32	0.19	0.23	0.40
Ficolin3 (µg/ml)	0.17	0.27	0.17	0.29	0.26	0.21	0.14	0.50	0.24	0.35	0.26	0.32

Spearman’s correlations (r_s_) between hepatic IR-index (a measure of hepatic insulin resistance) and serum levels of C4, C3 and lectin pathway initiators. Statistical significance assumed for values *P ≤ 0.05, **P ≤ 0.01, ***P ≤ 0.001. CON, control diet; HFO, high-fat overfeeding; NBW, normal birth weight individuals; LBW, low birth weight individuals; ALL, NBW+ LBW individuals.

### Association of peripheral insulin-resistance with serum levels of C4, C3 and lectin pathway initiators

The correlations of the different complement factors with peripheral insulin sensitivity (M-value) are shown in Table 4. When looking at the whole study population (n = 43), Ficolin-1 was negatively correlated to M-value after HFO (r_s_ = −0.38 P = 0.01). This finding persisted when analysing the NBW-subjects after HFO (r_s_ = −0.42 P = 0.03), but not in LBW subjects. In LBW subjects Ficolin-3 was negatively correlated to M-value, although only on the control diet (r_s_ = −0.50 P = 0.04). C4 and C3 did not correlate with peripheral insulin-resistance in the whole group or any of the subgroups regardless of the diet.

**Table 4:** Associations between M-value and serum levels of C4, C3 and lectin pathway initiators.

Parameter	ALL (n = 43)				NBW (n = 25)				LBW (n = 18)			
	CON		HFO		CON		HFO		CON		HFO	
	r_s_	p value	r_s_	p value	r_s_	p value	r_s_	p value	r_s_	p value	r_s_	p value
C4 (mg/ml)	−0.10	0.51	−0.14	0.36	−0.06	0.76	−0.32	0.11	−0.12	0.63	0.17	0.51
C3 (mg/ml)	−0.12	0.46	−0.16	0.31	−0.12	0.55	−0.29	0.16	−0.06	0.81	−0.03	0.90
MBL (ng/ml)	−0.11	0.48	0.08	0.63	−0.23	0.27	−0.02	0.93	−0.25	0.32	0.13	0.62
Ficolin1 (µg/ml)	0.13	0.40	−0.38	0.01^*^	0.04	0.85	−0.42	0.03^*^	−0.25	0.32	−0.30	0.22
Ficolin2 (µg/ml)	−0.29	0.06	−0.02	0.87	−0.12	0.56	−0.19	0.37	−0.13	0.60	−0,05	0,84
Ficolin3 (µg/ml)	−0.16	0.31	−0.24	0.13	0.23	0.26	−0.03	0.90	−0.50	0.04^*^	−0.32	0.21

Spearman’s correlations (r_s_) between M-value (an inverse measure of insulin resistance) and serum levels of C4, C3 and lectin pathway initiators. Statistical significance assumed for values *P ≤ 0.05, **P ≤ 0.01, ***P ≤ 0.001. CON, control diet; HFO, high-fat overfeeding; NBW, normal birth weight individuals; LBW, low birth weight individuals; ALL, NBW+ LBW individuals.

## Discussion

Insulin resistance has consistently been associated with a high serum level of C3 in a potentially causal manner^13–16^. However, the physiological mechanisms remain unclear. In this study we aimed to investigate whether insulin resistance induced by a high-fat diet might influence the complement system, and particularly circulating C3 level and complement activation. Our short term HFO intervention is relevant as it resembles commonly occurring feast periods in different cultures all over the world, and HFO over longer periods is one of the most important risk factors of obesity, insulin resistance and T2D.

We hypothesized that our short term HFO challenge would increase serum level of C3 and induce complement activation possibly affecting the development of insulin resistance. However, we found a significant decrease in serum levels of C4 and C3 alongside the development of insulin resistance in response to short term HFO. In line with these findings, we found a significant decrease in the activation capacity of all three complement pathways after HFO. To our knowledge, this is the first report to describe a short term HFO diet-induced decrease in circulating C4 and C3 in humans.

In contrast to our hypothesis of an increase in complement factors and complement activation during HFO, complement activation products C3bc and TCC and C3bc/C3 ratio decreased paradoxically in the whole group after HFO. Accordingly, our results do not directly support the idea that improper complement activation influences the development of insulin resistance in response to overly rich nutrition in humans at least in an acute setting.

**Figure 4:**
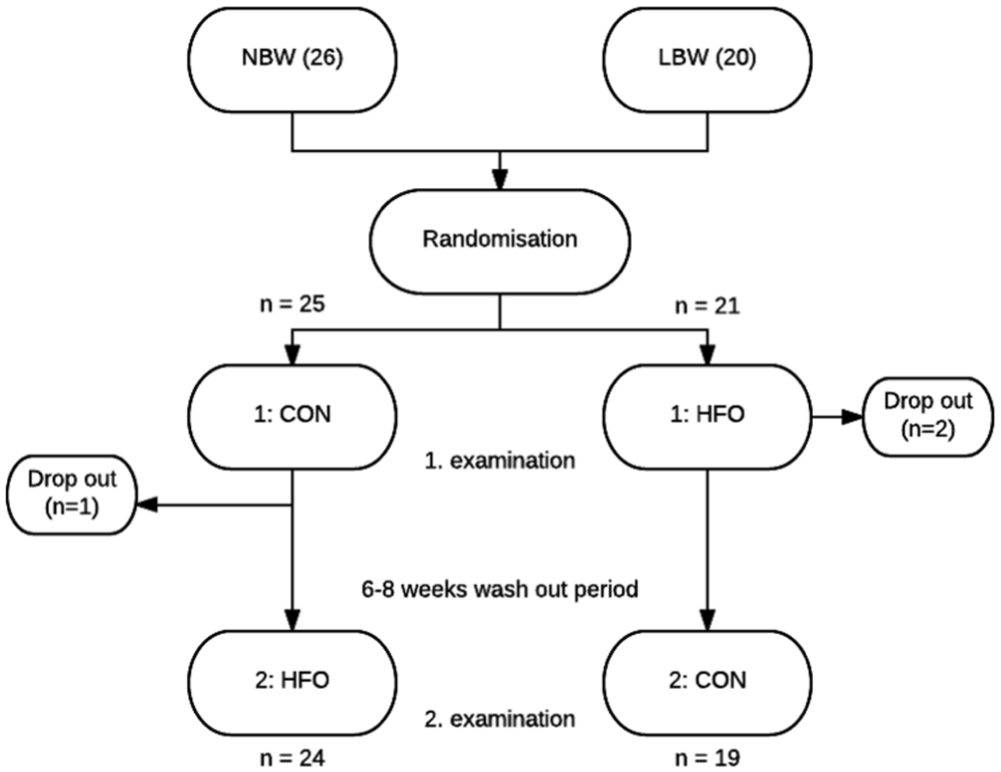
Study design. Flow chart showing the randomised crossover design of the study. The 46 participants were randomised to receive either the control diet before the first examination and high-fat overfeeding before the second examination or to receive the high-fat overfeeding before the first examination and the control diet before the second examination. The two study periods were separated by a 6–8 weeks wash out period. One participant experienced discomfort during the first clamp examination; why it was terminated and not repeated. This participant was excluded. Two participants did not complete the overfeeding intervention and were therefore excluded. CON, control diet; HFO, high-fat overfeeding; LBW, low birth weight; NBW, normal birth weight.

The randomised crossover design of this study (Fig. 4) allowed us to examine the reversibility of this complement dampening effect of short term HFO, by comparing the 24 subjects who were randomised to receive the control diet 6–8 weeks before the HFO diet with the 19 subjects who were randomised to receive the HFO diet before the control diet (Figs 2 and 3). Interestingly, the comparison showed that the impact of HFO on C4 was not significantly reversible after 6–8 weeks. C3bc and TCC increased significantly in the group receiving HFO before the control diet, indicating a reversible effect of HFO on complement activation. When looking at the impact of HFO on C3 there is a trend towards reversibility as well (Fig. 2D), and the same trend is seen for the impact of HFO on the functional complement activation capacity (Fig. 3). Thus our observations suggest that the complement dampening effect of short term HFO is in general a transient phenomenon.

There are several potential mechanistic explanations for the observed decrease in serum C4 and C3 levels in response to HFO. First, it is possible that HFO stresses the liver and reduce its ability to produce complement factors due to ectopic fat accumulation. We have previously shown that HFO induces hepatic insulin resistance and increases circulating liver enzyme ASAT, which is indeed an indication of a stressed liver^6, 7^. With the liver being the primary site for C4, C3, Ficolin-2, Ficolin-3 and MBL synthesis^21, 22^, a decrease in liver function could explain decreased circulating levels of these proteins and consequently a decreased capacity of complement activation. However, we did not find a decrease in the circulating levels of Ficolin-2, Ficolin-3, MBL or albumin also produced in the liver. Therefore our results do not indicate that short term HFO has a general impact on liver protein synthesis, and a diet-induced inhibition of hepatic biosynthesis specifically of C4 and C3 has to our knowledge not been reported previously.

Another explanation could be that hyperinsulinaemia in the overfed subjects promotes a down regulation of C4 and C3. Continuous insulin infusion has previously been shown to down regulate circulating C3 in severely burned children^23^, and insulin is well known to exhibit various systemic anti-inflammatory effects in humans including suppression of pro-inflammatory cytokines as well as induction of anti-inflammatory mediators^24–27^. A scenario could be that hyperinsulinaemia may influence transcription factors relevant for hepatic C4 and C3 synthesis.

These possible explanations of the decrease in C4 and C3 levels are not mutually exclusive, and irrespective of the underlying mechanisms, a key question is whether diet-induced lowering of complement proteins may be harmful if occurring in for instance elderly and more immunocompromised people and/or with longer exposure to overfeeding. Indeed, the complement system protects against invading microorganisms, and complement defects have various different consequences including recurrent bacterial infections, autoimmune manifestations and renal disorders, depending on the specific complement defect^28^. For instance, we cannot exclude the possibility that the changes in the gut microbiota during high-fat feeding as seen in mice and insulin resistant humans could be triggered by changes in the complement system^29^.

The LBW group was included in the study because of their known increased risk of developing insulin resistance and T2D^3–5, 7^. However, we did not find any difference between the NBW controls and the LBW subjects regarding circulating levels of C4, C3, C3bc, TCC or lectin pathway initiators on the control diet. Neither did we find any consistent differences between the two groups in the decrease of C4 or C3 levels, or in the complement activation capacity in response to HFO. Circulating C3bc and the C3bc/C3 ratio decreased significantly in the LBW subjects in response to HFO when compared to NBW subjects, where C3bc and the C3bc/C3 ratio remained unchanged after overfeeding. While this small difference between groups may have occurred as the result of a chance finding, we demonstrated that HFO did not induce complement activation detectable in plasma in any of the two groups. Thus, our data do altogether not support the idea that LBW individuals‘ predisposition to insulin resistance, T2D and cardiovascular disease may occur as a result of increased complement system activation.

Another novel finding of the current study is the selective association between plasma C3 levels and hepatic but not peripheral insulin resistance. We used the gold standard hyperinsulinemic euglycemic clamp technique enabling us to distinguish between hepatic and peripheral insulin resistance. Previous studies have reported associations between high circulating levels of C3 and insulin resistance using the more indirect homeostasis model assessment of insulin resistance (HOMA-IR)^13–16^. HOMA-IR predominantly reflects hepatic insulin resistance indirectly supporting our results. The LBW subjects did not show a significant association of C3 with hepatic insulin resistance after HFO, but an obvious trend was seen (r_s_ = 0.41 P = 0.09) and the lack of significance is possibly to be due to the small sample size.

Our finding that insulin resistance induced experimentally by short term HFO was not associated with any detectable complement activation in serum is an argument against the hypothesis that insulin resistance may be induced by complement activation. However, a limitation of our study is that we measured complement activation using ELISA on plasma. Over the last decades it has become clear that adipose tissue is a major source of complement products such as C3 and proximal components of the alternative pathway, factor B and factor D (adipsin)^30–32^. The role of complement in adipose tissue is only scarcely explored. Complement is thought to promote peripheral insulin resistance via stimulation of macrophage infiltration and activation in adipose tissue by the anaphylatoxins C3a and C5a. Activated adipose tissue macrophages are well known to produce pro-inflammatory cytokines, like IL-6 and TNF-∝, that inhibits the insulin receptor and thereby induce insulin resistance^10^. On the other hand C3 and its cleavage product C3a desArg are now recognized to have important roles in lipid storage and metabolism. C3a desArg, like insulin, stimulates FFA and glucose uptake in adipocytes, and it is generated in proportion to the total amount of C3^33^. Very recently the anaphylatoxins C3a and C5a have been shown to have insulin-like properties as well^20^. A recent study by Lo *et al*. showed that anaphylatoxin C3a improves insulin secretion from β-cells in diabetic mice^34^. Thus more and more evidence supports a dual role of the complement system in adipose tissue in relation to obesity, insulin resistance and T2D. It is therefore possible that local complement activation within the adipose tissue may affect insulin sensitivity of the adipose tissue and contribute to the reduced peripheral insulin resistance observed in the LBW subjects after overfeeding. Nevertheless, such a local change in complement activity may not be detected when performing ELISA on plasma, and thus further studies with direct assessment of local complement activation are needed before drawing to the conclusion that complement activation does not contribute to the development of high-fat-diet-induced insulin resistance.

In conclusion, we have shown that short term high-fat overfeeding induce a decrease in serum levels of C4 and C3 in healthy young men, affecting their complement activation capacity significantly. Birth weight associated risk of developing insulin resistance did not influence these findings. We have furthermore documented that serum level of C3 is positively associated with hepatic, but not peripheral insulin resistance in healthy young men. Further studies are required to understand the mechanisms underlying - as well as potential consequences of these findings.

## Research Design and Methods

### Study population

As previously described^6, 7^, 46 healthy male volunteers 23–27 years of age were recruited from the Danish Medical Birth Register. Twenty males were born with a low birth weight (BW ≤ 10th percentile) (2688 ± 269 g), and 26 age- and BMI-matched controls were born with a normal birth weight (BW between 50–90th percentile) (3893 ± 207 g). All were singletons and born at term in the area of Copenhagen. Exclusion criteria included family history of diabetes (in 2 generations), body mass index (BMI) greater than 30, and high physical activity level (>10 h of exercise per week). None of the participants had known illness or took medications known to affect the study outcome. Informed consent was obtained from all participants, and the protocol was in accordance with The Helsinki Declaration and approved by the ethical committee for Copenhagen County (KA-03129-gm).

### Study design

The data presented here are a part of a larger study investigating the impact of LBW and HFO on T2D pathophysiology and some data have thus been published previously, but are included here to provide the background of the current findings^6, 7^. All participants were examined twice in a randomised order; after a control period (CON) and after 5 days high-fat overfeeding (HFO). 25 subjects had their first examination after a control diet and 21 subjects had their first examination after a HFO diet (Fig. 4). Prior to each examination, were the participants standardized for 5 days with regards to diet, exercise and alcohol intake and the CON experiment was optimized even further by providing all foods (30% fat) for 3 days before the clamp examination. The HFO diet was provided to the participants and consisted of 60% fat and contained 50% more calories than individually required^7^. The requirement was multiplied by a factor of 1.5 corresponding to a low physical activity level. The two study periods were separated by a 6–8 weeks wash-out-period and subjects were asked to remain weight stable during this period. Participants were expected to eat all of the provided food, however to ensure compliance potential leftovers were collected, weighed and subtracted from the total energy intake. Two LBW participants did not complete the overfeeding intervention and one NBW experienced discomfort during the first clamp examination; therefore, it was terminated and not repeated. These 3 participants were therefore excluded.

### Investigation of insulin-resistance

Following the CON and the HFO period, insulin sensitivity and hepatic glucose production were measured by a 3-hour hyperinsulinemic euglycemic-clamp combined with a continuous infusion of tritiated 3-^3^H-glucose. In short, an insulin-infusion of 80 mU m^−2 ^min^−1^ was used throughout the clamp and to maintain euglycemia of 5 mmol/l. A variable infusion of glucose enriched with tritiated 3-^3^H-glucose was applied. The insulin-stimulated glucose disposal rate (M-value) and hepatic insulin resistance index (hepatic IR-index) were calculated as described previously^6, 7^.

### Serum concentration of C4, C3 and lectin pathway initiators

Serum concentrations of C4 and C3 were measured using an automated turbidimetric protein analyser (SPAPLUS®, The Binding Site group LDT, Birmingham, UK). The used antibodies were sheep polyclonal antibodies, raised against human C3c and human C4 respectively (The Binding Site group LDT, Birmingham, UK). MBL and ficolin serum concentrations were determined by established ELISA-based methods using antibodies produced in our own laboratories^35–38^. In short, microtiter plates were coated with one of the following monoclonal antibodies: anti-MBL (HYB-131-1), anti-Ficolin-1 (FCN166), anti-Ficolin-2 (FCN216), or anti-Ficolin-3 (FCN334), in phosphate-buffered saline (PBS). The plates were incubated overnight at 4 °C. Diluted serum samples were applied to the plates and incubated for three hours. A standard dilution series of pooled human serum with known concentration of MBL, Ficolin-1, Ficolin-2 and Ficolin-3 was added to each assay. Plates were incubated with biotinylated antibodies: monoclonal anti-MBL (HYB-131-1), polyclonal anti-Ficolin-1 (Hycult), monoclonal anti-Ficolin-2 (FCN219), and monoclonal anti-Ficolin-3 (FCN334) overnight at 4 °C. HRP-conjugated streptavidin was added to the wells and incubated for one hour. Plates were washed in PBS-tween between every step. At last o-phenylenediamine dihydrochloride (OPD) substrate solution containing H_2_O_2_ was added to the wells. Plates were developed for 15 min. Addition of sulphuric acid was used to terminate the enzymatic reaction and the optical density was measured at 490 nm (ELx808™ Absorbance Microplate Reader, BioTek, Vermont, USA).

### Assays for complement activation markers

The complement activation products C3bc and TCC were measured in EDTA-plasma samples by established ELISA-based methods using antibodies produced in our own laboratories^39–41^. In short, microtiter plates were coated with monoclonal antibodies: anti-C3bc (clone BH6) or anti-C9 (clone aE11) in PBS. The plates were incubated overnight at 4 °C. Diluted plasma samples were applied to the plates and incubated for one hour at 4 °C in the C3bc-assay and one hour at room temperature in the TCC-assay. A standard dilution series of pooled zymosan activated serum was added to each assay. Plates were incubated with biotinylated antibodies: polyclonal anti-C3c (DAKO, Q0368) or monoclonal anti-C6 (clone 9C4) for one hour. HRP-conjugated streptavidin was added to the wells and incubated for one hour. Plates were washed in PBS-tween between every step. At last plates were developed with TMB One (Kem-En-Tec Diagnostics) for 15 min. Addition of sulphuric acid was used to terminate the enzymatic reaction and the optical density was measured at 450 nm (ELx808™ Absorbance Microplate Reader, BioTek, Vermont, USA). A C3bc/C3-index was calculated, allowing us to normalize the C3bc concentration in relation to the total concentration of C3, so that variations in C3 concentration did not influence the assessment of the complement activation analysis.

### Functional capacity of the classical, alternative and lectin pathway

The complement activation capacity of the classical, alternative and lectin pathway was determined using an established semi quantitative ELISA-assay (WIESLAB® Complement System Screen COMPL 300, Euro Diagnostica, Malmö, Sweden). In brief: wells were precoated with specific activators of the classical, alternative and lectin pathway. Patient serum was diluted in different buffers containing specific inhibitors ensuring that only the respective pathway was activated. A positive control (PC) and a negative control (NC) were added to each assay. The plates were incubated with the diluted serum samples and diluted controls for one hour and then washed. A specific alkaline phosphatase labeled antibody detecting C5b-9 was added to all the wells. Plates were incubated for half an hour and subsequently washed. At last a phosphatase substrate solution was added to the wells and incubated for half an hour. The optical density was measured at 405 nm and the complement activation capacity in % was calculated as follows: (sample - NC)/(PC - NC).

### Statistics

All data were analysed for normality using Shapiro-Wilk normality test and appropriate statistical tests was applied. NBW and LBW individuals were compared using an unpaired t-test, or a Mann-Whitney test when data were not normally distributed. When looking at the effect of overfeeding a two-tailed paired t-test was applied on normally distributed data, while a Wilcoxon test was used on non-parametric data. To determine the association of peripheral and hepatic insulin-resistance with serum levels of C3, C4 and lectin pathway initiators we used the Spearman Rank correlation on all data. Statistical significance was assumed for P-values ≤ 0.05. All analyses were performed using GraphPad Prism 4, San Diego, California, USA.
